# Virtual Reality Applications for the Implementation of Domestic Respiratory Rehabilitation Programs for Patients With Long COVID and Post-COVID Condition: Scoping Review

**DOI:** 10.2196/52309

**Published:** 2024-05-31

**Authors:** Katharina Dalko, Hlynur Andri Elsuson, Ivonne Kalter, Max Zilezinski, Sebastian Hofstetter, Dietrich Stoevesandt, Denny Paulicke, Patrick Jahn

**Affiliations:** 1 Dorothea Erxleben Lernzentrum Medical Faculty Halle Martin-Luther-University Halle-Wittenberg Halle (Saale) Germany; 2 LichterSchatten Therapiezentrum GmbH Berlin Germany; 3 Health Service Research Working Group, Acute Care, Department of Internal Medicine Faculty of Medicine, University Medicine Halle (Saale) Martin-Luther-University Halle-Wittenberg Halle (Saale) Germany; 4 Institute for Clinical Nursing Science Charité - Universitätsmedizin Berlin Berlin Germany; 5 Department of Medical Education Akkon University of Human Sciences Berlin Germany

**Keywords:** long COVID, post-COVID, rehabilitation, virtual reality, implementation, respiratory, respiratory rehabilitation, scoping review, development, accessibility, support, physical, psychological, motivation, compliance, usability, COVID-19, COVID

## Abstract

**Background:**

Due to a high number of patients affected by long COVID or post-COVID condition, an essential step to address the long-term effects of COVID-19 lies in the development and implementation of flexible and accessible rehabilitation programs. Virtual reality (VR) technologies offer the potential to support traditional therapies with individualized at-home programs.

**Objective:**

This study aims to provide an overview of existing scientific evidence on the development and implementation of VR-assisted respiratory rehabilitation programs for patients with long COVID and post-COVID condition and to synthesize the results.

**Methods:**

We conducted a scoping review of studies from 6 databases. PubMed, CINAHL, Cochrane, ScienceDirect, Web of Science Social Sciences Citation Index, and PEDro were searched using an exploratory search strategy. The search, which was last updated in February 2024, included peer-reviewed studies on immersive VR applications providing respiratory rehabilitation programs for patients with chronic obstructive pulmonary disease and long COVID or post-COVID condition. Exclusion criteria were studies in clinical or inpatient settings, telemedicine, nonimmersive VR applications, and gray literature. Nine publications were included in this review. Findings were extracted and summarized from the studies according to the JBI (Joanna Briggs Institute) method and thematically categorized. Topics covered were study characteristics, physiotherapeutic concept, clinical parameters, as well as usability and acceptability.

**Results:**

The 9 publications included in the qualitative analysis were published in 2019-2023. Eight empirical studies were included: 4 followed a mixed methods design, 3 were qualitative studies, and 1 followed a quantitative method. One scoping review was included in the data analyses. Four of the included studies were on patients with chronic obstructive pulmonary disease. The 9 studies demonstrated that VR-supported respiratory rehabilitation programs result in positive initial outcomes in terms of physical as well as psychological parameters. Particularly noteworthy was the increased motivation and compliance of patients. However, adverse effects and lack of usability are the barriers to the implementation of this innovative approach.

**Conclusions:**

Overall, VR is a promising technology for the implementation of individualized and flexible respiratory rehabilitation programs for patients with long COVID and post-COVID condition. Nevertheless, corresponding approaches are still under development and need to be more closely adapted to the needs of users. Further, the evidence was limited to pilot studies or a small number of patients, and no randomized controlled trials or long-term studies were part of the study selection. The included studies were performed by 4 groups of researchers: 3 from Europe and 1 from the United States.

## Introduction

### Background

In the wake of the COVID-19 pandemic, the rehabilitation of patients in the postacute phase of this disease is an important measure to address the long-term effects since a significant number of patients experience the condition commonly known as long or post-COVID [1]. This condition is characterized by symptoms that persist or develop after the acute phase of infection—starting from the fourth week after infection—and that cannot be explained by an alternative diagnosis [2,3]. The number of affected patients varied in the studies depending on the methodology, symptoms, as well as the population included in the analysis. Although studies provide heterogeneous results and case numbers, they show that a significant number of patients are affected by persistent symptoms after a COVID-19 infection. Patients who report an impact on everyday functioning up to 3 months after testing negative account for 10%-50% of the study participants [4]. According to a study by Peter et al [5] conducted in 2020 and 2021, including 11,710 patients from Germany, 28.5% of the patients reported persistent symptoms for 6-12 months after infection with COVID-19. That study further estimated that at least 6.5% of the adult patients in the general population who had recovered from COVID-19 infection were affected by long-term symptoms such as fatigue, dyspnea, neurocognitive impairments, and chest pain [5]. As per the World Health Organization, in the European region approximately 20% of the patients developed symptoms mentioned above continuing for at least 3 months after recovery according to a meta-analysis [6]. In addition, psychological symptoms such as anxiety and stress can have a negative impact on the quality of life caused by the abovementioned long-term effects [4,7].

After the treatment of acute symptoms is completed, patients need postacute rehabilitation, where physical therapy plays an important role in the treatment of lung-specific symptoms. For the best treatment possible, outpatient programs as well as solutions for the implementation of therapy programs in the home environment have to be established [8,9]. Pulmonary therapy approaches for chronic diseases such as chronic obstructive pulmonary disease (COPD) designed to normalize respiratory function have been well-established and are guiding the development of therapies for patients with long COVID or post-COVID condition. Therapy approaches include mobilization exercises, endurance, as well as strength training [10-14]. Since many patients experience psychological symptoms from the effects of impaired respiratory function, it is necessary to guide, counsel, and train patients in the use of appropriate strategies and coping skills when acute respiratory distress occurs [11].

Although the number of people affected by long and post-COVID symptoms remains high even as the pandemic situation continues to ease, there is still insufficient knowledge about the disease and a shortage of specialists and therapy programs [4,15]. In addition, the physical limitations of patients further impact their access to traditional physical therapy services. One way to address the shortage of adequate programs is to develop digital therapy solutions and assistive devices that are applicable in a home setting and can be individually applied without the constant supervision of specialist staff.

Initial findings suggest that digital approaches enable a more accessible implementation of therapies in the home environment and, at the same time, can contribute to increased motivation and adherence to therapy on the part of patients [16]. Previously established digital applications, for example, for patients with COPD have led to an increase in the quality of life, particularly with regard to emotional control and reduction in fatigue and dyspnea [17]. Virtual reality (VR) technologies are a solution to implement individual and flexible physical therapies in virtual space through the virtual representation of therapeutic measures and therapy situations. Approaches to integrate immersive VR applications already exist in various areas of rehabilitation as well as in psychotherapy, for example, to alleviate respiratory symptoms [18,19] and reduce anxiety and stress [20]. However, the implementation of respiratory therapy approaches for the home environment and especially programs for the target group of patients with long COVID/post-COVID condition are still under development. Further, a comprehensive review of the initial evidence on the development and implementation of appropriate VR applications does not yet exist.

### Objectives

The aim of our literature review was to (1) obtain an overview of the findings in international research regarding VR-based respiratory rehabilitation programs for patients with long COVID/post-COVID condition and (2) obtain criteria for the development and implementation of respective VR applications for the home environment. Our scoping review addresses the research question: what scientific evidence exists on the development and implementation of VR-assisted rehabilitation programs for patients with long COVID/post-COVID condition that are implementable in a home setting? The selection and analysis of the studies were based on the following subquestions:

1. Which guidelines exist for the design of VR respiratory rehabilitation programs?
2. What are the enabling aspects? What are the barriers to implementation?
3. What clinical outcomes have been reported?

## Methods

### Study Design

The methodological approach of the JBI (Joanna Briggs Institute) method according to von Elm et al [21] was adopted as the basis of this scoping review to give a broad overview on the existing findings and identify established criteria in international research for the implementation of VR-assisted rehabilitation programs for patients with long COVID/post-COVID condition. Since only a small number of studies was expected regarding the patient group and the focus was on respiratory rehabilitation programs, conditions with a comparable symptomatic spectrum—such as patients with COPD—were also included. Results will be reported using the PRISMA-ScR (Preferred Reporting Items for Systematic Reviews and Meta-Analyses extension for Scoping Reviews) guidelines (Multimedia Appendix 1). In preparation for this, a review protocol was developed but not published or registered.

### Search Strategy

A sensitive database search was conducted using the databases PubMed, CINAHL, Cochrane, ScienceDirect, Web of Science Social Sciences Citation Index, and PEDro. The search was last updated in February 2024 in order to incorporate newly released studies. According to the search components—population, concept, and context—search terms were applied using Boolean operators, truncations, and proximity operators (see Textbox 1 and Multimedia Appendix 2).

**Search string using the example of the search in PubMed.**

(((covid-19[MeSH Terms]) OR (respiratory*[Title/Abstract])) OR (pulmonary*[Title/Abstract])) AND (Rehabilitation[Title/Abstract]) AND (VR[Title/Abstract] OR virtual reality[Title/Abstract])

The identified studies were merged in the web-based tool “rayyan” [22] and screened by titles, abstracts, and full texts in regard to the research question, which was conducted independently by 3 researchers. Additionally, the reference lists of all the publications included in the full text screening were searched for further evidence. The inclusion and exclusion criteria for the studies were decided by team consensus (KD, IK, and HAE).

### Study Selection

All types of studies published in the period between 2012 and 2023 that were available in English or German and provided with an abstract were included. The following inclusion and exclusion criteria were applied (see Textbox 2).

**Inclusion and exclusion criteria for the studies in this review.**

**Inclusion criteria**

- Studies on immersive virtual reality applications providing respiratory rehabilitation programs, including breathing exercises, physical training, education, and programs introducing psychological counseling such as stress reduction for the home environment
- Studies including patients with long COVID, post-COVID condition, or chronic obstructive pulmonary disease
- Peer-reviewed empirical studies
- Mixed methods, qualitative, and quantitative studies

**Exclusion criteria**

- Clinical inpatient setting
- Telemedicine
- Virtual reality applications that are nonimmersive (applications for personal computers, augmented reality, etc)
- Gray literature (conference papers, opinion papers, study protocols)

### Data Extraction and Synthesis

The characteristics of the identified studies were mapped in a preconsented data form (KD) and summarized narratively. Three researchers (KD, IK, and HAE) derived evidence on the development and implementation as well as the clinical outcomes of the studies concerning VR-based rehabilitation programs from the literature included in this analysis. The aspects identified were then categorized thematically, while study results covered the areas of study characteristics, physiotherapeutic concept, and outcomes in terms of clinical parameters as well as usability and acceptability aspects and were clustered and summarized according to the JBI method [21]. The categorization was then discussed within the research team.

## Results

### Overview

**Figure 1:**
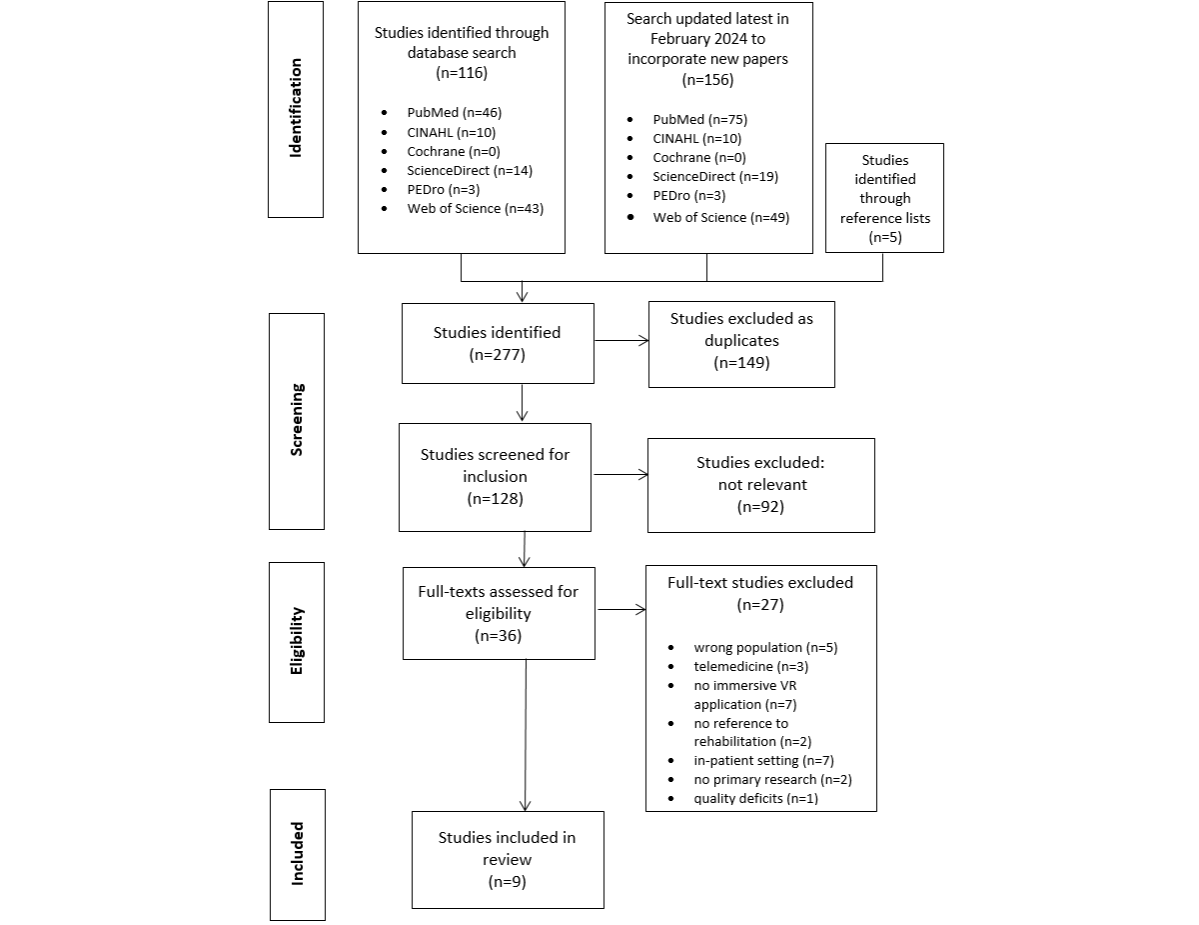
PRISMA-ScR (Preferred Reporting Items for Systematic Reviews and Meta-Analyses extension for Scoping Reviews) flowchart of the study selection process.

After duplicates were removed, 128 identified abstracts according to the above listed criteria were independently reviewed by 3 authors (KD, IK, and HAE). A full-text screening of the resulting 36 publications led to 9 studies, which were included in our review. Figure 1 shows the process of study selection in a PRISMA flowchart.

### Characteristics of the Included Studies

The included studies were published between 2019 and 2023. The majority of the studies [23-28] were published since 2022. Six empirical studies were conducted in Europe and were limited to the Netherlands, United Kingdom, and Slovakia [19,25-29]. Further, 2 publications that refer to the same study conducted in the United States were included [23,24]. Four studies followed a mixed methods design [19,23,25,27], 3 studies were qualitative studies [24,28,29], and 1 study applied only quantitative methods [26] (Table 1). The data analyses included 1 coping review: in 2020, Colombo et al [30] reported VR applications and exergaming for pulmonary rehabilitation of patients with COPD. Both immersive and nonimmersive approaches were included. However, an assessment of the results with regard to the quality of the included studies was not performed. There was no systematic review on immersive VR rehabilitation programs for home settings and targeting patients with long COVID/post-COVID condition.

**Table 1:** Full list of the publications included in this scoping review.

Study, year	Objective	Study design	Population type	Sample size, n
Colombo et al [30], 2020	Literature review exploring findings on virtual reality and exergaming applications for pulmonary rehabilitation with focus	Scoping review	Patients with COPD^a^	N/A^b^
Gabriel et al [23], 2023	Qualitative evaluation of the feasibility of a pulmonary rehabilitation program, including educational content for patients with COPD	Mixed methods study	Patients with COPD	18
Gabriel et al [24], 2023	Qualitative evaluation of a pulmonary rehabilitation program for patients with COPD	Qualitative study	Patients with COPD	9
Groenveld et al [25], 2022	Evaluating self-administered virtual reality exercises at home for post–COVID-19 condition	Mixed methods study	Patients with post-COVID	48
Jung et al [19], 2020	Investigating whether virtual reality provides a credible alternative to traditional pulmonary rehabilitation programs and improves compliance among patients with COPD	Mixed methods study	Patients with COPD (Medical Research Council Dyspnea scale 4-5)	10
Lacko and Ruzický [26], 2022	Analyzing the use of virtual reality devices to rehabilitate patients in a controlled outpatient environment as well as in the home environment	Quantitative study	Patients with long-term COVID or post-COVID syndrome	16
Moorhouse et al [29], 2019	Evaluating a virtual reality pulmonary rehabilitation for patients with COPD	Qualitative study	Patients with COPD (Medical Research Council Dyspnea scale 4-5)	10
Ruzicky et al [27], 2022	Investigating the prevention, diagnosis, and treatment of patients after COVID-19 while using artificial intelligence and virtual reality in combination with traditional approaches to patient rehabilitation	Mixed methods study	Patients with post-COVID	10
Smits et al [28], 2022	Developing an evidence-based “Guidance ethics in context” for virtual reality development	Qualitative study	Patients with long COVID (n=20), physical therapists (n=15)	35

^a^COPD: chronic obstructive pulmonary disease.

^b^N/A: not applicable.

### Synthesis of Results

The following section presents a synthesis of evidence from the included studies as the result of the qualitative analysis. The narrative description is based on the aspects of study characteristics, rehabilitation program, technical implementation, and evaluative and clinical outcomes.

#### Study Characteristics

The studies were first divided into 2 groups. The English research group led by Jung et al [19] and Moorhouse et al [29] and a US-American research group [23,24] tested VR applications designed for patients with COPD. They refer to the significance of continuous pulmonary rehabilitation, education of patients regarding the characteristics of their disease, and useful behavioral interventions. They also reported low compliance due to depression related to the condition, low awareness of the potential therapeutic approaches among patients, and lack of knowledge about the benefits of continuous therapy for chronic conditions. Immersive VR applications are intended to create an innovative motivating rehabilitation approach in this context [19,23,24,29]. The other publications refer to the development or evaluation of immersive digital rehabilitation programs for patients with long COVID or post-COVID condition [25-28]. Although the post-COVID condition, as already described in the introduction, refers to persistent symptoms from 4 weeks after a COVID-19 infection [26,27], other studies do not distinguish between long COVID and post-COVID condition at all [25]. Long COVID is usually described as a long-term consequence of infection with SARS-CoV-2 with various physical, psychological, and cognitive symptoms [25-28].

Ruzicky et al [27] collected data on symptoms and severity of the disease during COVID-19 infection and after recovery among a group of students and professors via a questionnaire. Based on the results, which included fatigue, fever, shortness of breath, and depression as common post-COVID symptoms, 2 groups of patients were included in the study. Ten patients had mild muscle pain and shortness of breath after mild exertion and another 6 patients were included who reported severe muscle pain along with shortness of breath [27]. None of the studies required a medical diagnosis or the submission of test results to verify if participants were actually infected with COVID-19; instead, studies relied solely on patient reports. Moreover, none of the studies provided further information regarding the characteristics of participants apart from gender and age or the cultural diversity of populations included. Studies have characterized VR as a way of facilitating access to therapeutic measures in the context of the pandemic [25,27]. In addition, 2 studies implemented individualized and multimodal therapy programs, which address both physical and psychological factors such as stress and anxiety [25,28]. Gabriel et al [23,24] further implemented educational content regarding pulmonary rehabilitation as part of the VR program [23,24].

#### Collection of Evaluative Data and Assessment

All the studies mentioned dealt with the evaluation of pulmonary VR rehabilitation applications by patients with regard to technology acceptance, usability, and criteria for implementation [19,23-29]. Only 1 publication also included the viewpoint of physical therapists in the assessment [28]. The majority of the studies used interview procedures as the qualitative data collection method [19,24,27-29]. Focus groups in which those affected by COPD were able to discuss their experiences in using the VR application were also used in this context [19,29]. The quantitative survey instruments used in the studies by Jung et al [19] and Groenveld et al [25] included both standardized questionnaires and assessments of physical performance. The questionnaires included the Chronic Respiratory Disease Questionnaire, Patient Health Questionnaire-9 [19], and the 11-point Borg Scale [25], as well as questionnaires regarding psychological and cognitive factors such as Generalized Anxiety Disorder-7 items [19], Hospital Anxiety and Depression Score, and Cognitive Failure Questionnaire [25]. Gabriel et al [23] further applied questionnaires to assess the usability of the application such as the System Usability Scale [23]. Notably, Groenveld et al [25] collected physical performance parameters. These include the 6-Minute Walk Test, Timed Up and Go Test, or 30-Second Chair to Stand Test. In addition, sensors such as smart bracelets (heart rate, pedometer, hand movements, sleep cycles) and pulse oximeters were used to measure the progress of therapy in the studies [27]. Overall, clinical outcomes (physical as well as psychological) and the evaluation of the application used in terms of acceptance and usability were analyzed in the studies.

#### Rehabilitation Program

The physiotherapy programs in the studies described in Table 2 primarily involve respiratory physiotherapy aimed at improving patients’ functional ability [19,23-26,28,29].

**Table 2:** Characteristics of the rehabilitation programs evaluated in the studies included in this review.

Study, year	Type of training	Length of training	Setting of training	Virtual scenario
Gabriel et al [23], 2023	Physical training (pulmonary rehabilitation), educational content	Not reported	Home setting	Custom-made minigames and multiple choice
Gabriel et al [24], 2023	Physical training (pulmonary rehabilitation)	Not reported	Home setting	Custom-made minigames
Groenveld et al [25], 2022	Physical training, cognitive training, psychological exercises (meditation), independent training: self-management by patients	6-week trial	Home setting	Custom-made applications (minigames) for different exercises
Jung et al [19], 2020	Educational material, physical training (traditional pulmonary rehabilitation + focus on lower extremity), independent training: self-management by patients	8-week trial	Home setting, remotely supervised by health practitioners	Digital avatar
Lacko and Ruzický [26], 2022	Breathing exercises, physical training (upper limb), cognitive training	6-10 weeks	Diverse, remotely supervised by health practitioners	Digital avatar, photorealistic environments
Moorhouse et al [29], 2019	Physical training (pulmonary rehabilitation), educational material, independent training: self-management by patients	8-week trial	Home setting	Digital avatar
Ruzicky et al [27], 2022	Breathing exercises, physical training (upper limb)	Minimum of 3-4 weeks trial up to more than 5 months	Diverse, remotely supervised by health practitioners	Digital avatar, photorealistic environments
Smits et al [28], 2022	Physical training, cognitive training, psychological exercises (meditation), independent training: self-management by patients	6-week trial	Diverse	Custom-made applications (minigames) for different exercises

The focus of the studies was on various aspects such as relief of respiratory distress [19,23,24], rehabilitation of the upper limbs [26,27], and strengthening of the respiratory support muscles [19,29]. For this purpose, physiotherapeutic programs consisting of endurance, strength, and respiratory training were implemented. In addition, mental rehabilitation in light of the impact of the disease [28] as well as long-term goals such as feeling confident leaving the house [19] and quality of life [29] are addressed. Three studies also included exercises to improve cognitive skills [25,26,28]. Ruzicky et al [27] claimed that they addressed prevention, diagnosis, and treatment after COVID-19 but did not provide any further details on that. The length of the therapy programs ranged from 6 to 10 weeks [19,25,26,28,29]. Only in the study of Ruzicky et al [27], a minimum duration of 3-4 weeks was indicated, while in individual cases, the rehabilitation may last several months. In each case, the training program was designed for implementation in the home setting [19,23-29], but half of the publications also reported hybrid use cases within the study. For example, patients were able to try out the VR application in a therapy practice.

Moorhouse et al [29] as well as Jung et al [19], Smits et al [28], and Groenveld et al [25] implemented respiratory rehabilitation measures that were applied fully independently by patients. However, Ruzicky et al [27], Lacko and Ruzický [26], and Jung et al [19] implemented measures to monitor exercise progress. Patients were remotely assisted in setting up the program, for example, via telerehabilitation methods, while the exercises themselves were then performed independently [26,27]. Jung et al [19] focused on ensuring patient safety in their study on patients with COPD. For this reason, data such as heart rate and oxygen saturation were continuously measured in order to be able to intervene in the event of respiratory distress [19]. The selection of exercises was either defined by the patients themselves [29] or discussed with the therapist and adjusted to the requirements of the patients [26-28]. Ruzicky et al [27] and Lacko and Ruzický [26] specified that they included age, gender, and personality type in the selection process but did not specify what the criteria for the personality type is referring to and how age and gender influence the selection of the program.

The interdisciplinary research team consisting of researchers, medical doctors, physical therapists, designers, and VR developers as part of the study by Smits et al [28] developed a toolkit with resources and games. The program consisted mainly of pre-existing games and apps for physical, mental, and cognitive rehabilitation [28]. Three studies stated that a 3D avatar guides the exercises in the immersive VR environment [19,26,29]. Thereby, only 2 studies (from the same research team in Slovakia) chose a realistic representation of the avatar and environment [26,27]. Most commonly used VR headsets were Oculus Quest 2 [23,24,26,27] and Oculus Quest [25,28]. Other headsets used were Pico Interactive Goblin [19] and HTC VIVE Pro EYE [26]. Only Moorhouse et al [29] did not specify the headset used in their research.

#### Usability and Acceptance

VR-assisted digital respiratory rehabilitation was found to be generally acceptable and feasible in the reviewed studies. In this context, both the enabling factors and barriers for the development and implementation of corresponding applications could be derived from these studies. The benefits of virtual therapy include the aspects of immersion, motivation, as well as autonomy, flexibility, and the possibility of monitoring by therapists. In contrast, barriers include the initial adverse effects related to VR technology and the technical problems and lack of accessibility or usability of the VR applications.

#### Enabling Factors

In general, study participants described the tested applications as easy to use and enjoyable [19,23-25,28-30]. Studies in the context of respiratory rehabilitation of patients with COPD, in particular, also addressed the immersion in a virtual world, which among other things represents a distraction from the disease. In addition, the avatar guiding the exercises was considered as a social element to a certain extent [19,30]. VR is also considered easier to apply compared to traditional options such as instructions from printed material and booklets for the home environment [29]. Patients with COPD as well as patients with long COVID described the VR application as engaging and pleasurable [23,24,29]. They emphasized experiencing increased motivation to engage in therapeutic measures due to the stimulating or even calming nature of the virtual world, depending on the exercise [19,23-26,29,30]. The increased motivation leads to an increased frequency in usage [19,26,27]. Additionally, Smits et al [28] concluded that the gamification of exercises, in particular, contributes to the motivation of patients. Gamification refers to design elements that reproduce game elements and logics. This includes, for example, the integration of exercises into a game environment, earning scores through correct performance, and competitive approaches such as playing against each other.

Groenveld et al [25] distinguished between users according to their age and found that the duration of VR application increases with age. A possible explanation could be older persons’ lesser familiarity with VR technologies because of which they are slower in navigating through the application and they lose interest in the interactive environment and the immersion less quickly [25]. Other studies reported patient groups without any previous experience with VR technology showing difficulties at the beginning of the program [26,27]. Lacko and Ruzický [26] and Ruzicky et al [27] also address the so-called “WOW-effect” in their study, describing first-time users’ initial curiosity and great interest in VR [26,27]. However, according to this logic, boredom could also set in after a certain time of using the rehabilitation measure. In 2 studies, comments from study participants, including those who dropped out early, also indicated this same issue [25,26]. The digital program was described as boring, and doubts were expressed about the usefulness of the therapy [25].

Finally, the flexibility and autonomy in the implementation of VR therapy measures is emphasized in the reviewed studies [19,23,24,28,29]. This includes the feasibility of rehabilitation independent of time or location restrictions [23,24,28]. Jung et al [19] concluded that VR reduces the barriers for compliance by increasing the accessibility of rehabilitation programs, which are applicable in the home environment [19]. Patients mentioned that implementation in the home environment, in particular, can contribute to a feeling of comfort and security. In addition, patients reported that monitoring by therapists also gave them confidence [19,23,24]. Nevertheless, VR is perceived as a way to complement traditional therapies offering the advantage of social contact, which VR applications cannot fully compensate for [29].

All rehabilitation programs were customized for the patients in question. However, a distinction was made whether the program was determined by the therapist [19,26,27,29] or by the patients themselves [28]. Smits et al [28] also found that the ability to adapt the therapy to one’s own level of rehabilitation and to select exercises individually offered a high added value and contributed to patients’ autonomy. Likewise, Jung et al [19] and Moorhouse et al [29] corroborated the same findings in their evaluations.

#### Barriers

In addition to the benefits of VR-assisted respiratory therapy, barriers to VR implementation were described in these studies. First, the potential adverse effects of VR therapy are motion sickness or overextension due to immersion in an interactive virtual environment [25,28]. Motion sickness is a condition characterized by symptoms of nausea, vomiting, and dizziness caused by conflicting sensations related to motion. Motion sickness is commonly experienced when using VR devices, as the immersive nature of VR can create a sensory conflict between the visual input of a virtual environment and the lack of corresponding physical movement [31]. Further, dizziness, headache, or neck pain were among the most frequent reasons for patients to discontinue the studies [25,28]. Second, immersion can also cause anxiety through a realistic representation of an environment that does not match one’s own setting. For example, some patients were afraid of falling while performing the exercises [28]. Third, the VR application was sometimes perceived as overwhelming [25,28]. Smits et al [28] stated the assumption that cognitive impairment as part of long/post-COVID condition may also make the use of VR difficult or impossible for certain patients. This also means that VR-assisted therapy programs should be used individually depending on the condition of each patient. Smits et al [28] therefore recommend a close physiotherapeutic supervision, tracking of training sessions, as well as the monitoring of vital signs. Furthermore, the headset was felt to be too heavy [19,23,29,30].

Studies with a focus on the technical user-friendliness of the application provided information on how to improve the tested applications [26,27,29]. These included, in particular, information on the navigation of the programs (ie, pause or fast forward button [29], one-click solution to start program [26,27]), which states that the technical accessibility and intuitive environment that allows handling even by less technically experienced people, are of great importance. In addition, some studies recommend providing the virtual environment with a simple graphical user interface [26,27] and using clear instructions [30] to facilitate use. Smits et al [28] point out that lack of usability can not only prevent usage and acceptance but also influence the results of studies aiming toward the evaluation of efficacy of programs. An appropriate design could also reduce the abovementioned adverse effects. Therefore, an interdisciplinary design process is recommended to ensure usability [28]. Furthermore, therapists will need proper training and logistical support in regard to the use and implementation of respective VR technology to adequately supervise the training of patients [28].

#### Clinical Outcomes

In addition to usability and acceptance assessments, all studies listed here also collected clinical parameters for the evaluation of respiratory rehabilitation. These included both physical and psychological outcomes. In regard to the respiratory function and fatigue, an overall improvement was reported in 3 studies [19,25,27]. Jung et al [19] reported that outcomes of female participants with regard to dyspnea and fatigue were even better than the results from male participants. In addition, a significant increase in patients’ physical abilities such as strength and mobility was also observed [19,27-29]. Groenveld et al [25] found significant improvements in the 6-Minute Walk Test, grip strength, and 30-Second Chair to Stand Test. Ruzicky et al [27] mentioned that the program focused on developing upper limb mobility and cognitive skills through interactive tasks in the VR environment. However, information on the exact content of the tasks is lacking.

Surveys of the health-related quality of life and the Positive Health questionnaire and 12-Item Short Form Survey showed significant improvement in the quality of life in patients with COPD and patients with long COVID [19,25,29]. Patients felt fitter and were more likely to participate in social activities [19,29]. In the study of Groenveld et al [25], the improvement occurred already after 6 weeks. Further, stress and anxiety were reduced during the rehabilitation [19,25,29]. However, this was partly only true for patients who used specific mental health applications [25]. Participants also felt more confident in dealing with their own disease and in everyday tasks [19,28,29]. Some patients said that they were more mindful of their own health as a result of the program, making time for meditation on their own and setting preventive boundaries in their daily lives [28]. These experienced benefits of VR therapy as well as increased patient motivation also led to improved compliance [19,26-29].

## Discussion

### Principal Findings

The results of the reviewed studies show internationally available and initial evidence with regard to the development as well as the feasibility of respiratory VR rehabilitation for patients with long COVID in particular. The topic addressed in this review is a very new field of research. Approaches to VR therapy for other therapeutic needs such as for patients who had a stroke or Parkinson disease have already been implemented in the past 15 years [25,26,29]. However, lung-specific virtual physiotherapy appears to be still under development even in regard to the condition of COPD, which is already well-studied. The reported results are promising for VR applications, as the tested applications were described as enjoyable, pleasurable, and motivating when correctly introduced [19,23-27,29]. Furthermore, they can offer a more flexible rehabilitation program without restrictions of time and location [19,23,24,28,29]. Nevertheless, the studies also showed that the implementation of VR therapy interventions cannot generally be considered appropriate for every patient or in every setting because some patients could not complete the training due to motion sickness [25,28], and VR poses various hurdles in terms of the digital literacy of patients and therapists [23,24,28].

### Comparison With Prior Work

A previous study that is the most similar to ours is the comprehensive review of VR for pulmonary rehabilitation by Pittara et al [32] in 2023. They offer a broader overview of VR applications in pulmonary rehabilitation by including all types of VR experiences, ranging from nonimmersive to fully immersive, and various populations, including healthy individuals as well as patients with COPD and asthma. In comparison to Pittara et al [32], our review takes a more focused approach by specifically filtering publications to include only immersive VR experiences within pulmonary rehabilitation programs for outpatients. The main strength of our review therefore lies in its clearly defined goals to provide an overview of immersive VR experiences for outpatients experiencing long COVID and COPD. Our review highlights the findings and shortcomings of existing research specifically in relation to the implementation of home-based rehabilitation programs for the target group. In particular, the hurdles of digital literacy identified for implementation at home and the need for training to ensure adequate use and guidance can be highlighted in this regard [27-29].

Our study findings also show the necessity to include the needs and prior knowledge of the target groups in the development of appropriate therapies. Only when appropriate programs achieve added value (eg, through individualized programs, monitoring) and are at the same time easily implemented, they can be applied in practice. The approaches adopted to involve patients in the evaluation of the applications, in terms of usability and acceptance, have shown that patients can provide important information for the development and implementation of VR-supported therapies [19,28,29].

Participatory approaches to technology development, which involve patients already during the development of applications and therapy programs, could help to adapt the applications even more precisely to the needs and requirements of users. The World Health Organization, for instance, recommends a patient-centered development of rehabilitation measures, digital services, and devices in order to support the self-care competence, especially of patients with long/post-COVID condition [4]. Regarding the novelty of the postacute condition, the involvement of patients seems even more crucial because researchers, practitioners, and patients are still undergoing a learning process on how to address and manage the symptoms reported [15]. Therapists, who are to integrate the applications into their therapy services and train the patients in their use, can also provide concrete information on their feasibility in practice. Of the studies analyzed in this review, however, only 1 study design included therapists [28].

### Limitations

Although this scoping review was supported by steps, including refinement of the protocol through team discussion, blinded searching, and selection of papers by 3 researchers, several limitations have to be mentioned. First of all, limitations in the scope of the reported study results must be explained, as only 4 groups of researchers (from Slovakia, the Netherlands, United States, and United Kingdom) were involved in the studies analyzed, which represents quite a Eurocentric perspective. This scoping review was limited to studies published in English and German—the spoken languages of the researchers in this scoping review. However, the scoping review method and the explorative search strategy were deliberately chosen in order to be able to explore an overview of the topic while maintaining the focus of the research, and a critical evaluation of the studies was not intended. The second important aspect to note is that study designs implemented by the included studies and the quality in regard to scientific standards were very heterogeneous. Due to the diverse applied assessments and questionnaires, the comparability remains limited. Furthermore, evidence is still limited to pilot studies or a small number of patients, and no long-term studies or randomized controlled studies could be integrated—only cross-sectional surveys.

### Conclusion

The results of this scoping review show that VR applications are well accepted by users, especially due to their flexible and individual applicability. Particularly mentioned by patients was the possibility of individualizing training plans and schedules as well as monitoring functions for remote monitoring by therapists. The implementation of rehabilitation measures in a playful, immersive setting contributed to motivating patients and increasing their compliance in respective studies. Initial feasibility studies also show an improvement in physical performance as well as psychological parameters such as confidence in managing the disease and quality of life. At the same time, hurdles arise with regard to the technical feasibility of VR therapies. Virtual applications must be as accessible and easy to use as possible so that patients without prior knowledge can also benefit from the therapy options. Furthermore, scientific research has to further develop empirical reliable findings for the sustainable long-term implementation of support programs for patients with long/post-COVID condition in the years ahead. In particular, the question on how to implement these findings into practice with regard to financing, further education of therapists, technical support, and the alignment of traditional and innovative autonomous approaches to therapy have to be a priority.

## Appendix

Multimedia Appendix 1 PRISMA-ScR (Preferred Reporting Items for Systematic Reviews and Meta-Analyses extension for Scoping Reviews) checklist.

Multimedia Appendix 2 Search strategy.

## Abbreviations

COPD: chronic obstructive pulmonary disease
JBI: Joanna Briggs Institute
PRISMA-ScR: Preferred Reporting Items for Systematic Reviews and Meta-Analyses extension for Scoping Reviews
VR: virtual reality
