# Supplementary material for: Virtual Reality Applications for the Implementation of Domestic Respiratory Rehabilitation Programs for Patients With Long COVID and Post-COVID Condition: Scoping Review
Source: JMIR Serious Games. 2024 May 31;12:e52309. doi: 10.2196/52309 (PMC11179016; doi:10.2196/52309)
Supplement: Multimedia Appendix 2 [file games_v12i1e52309_app2.docx]

**Search strategy (search February 2024)**

**Pubmed**

Results: 75

(((covid-19[MeSH Terms]) OR (respiratory*[Title/Abstract])) OR (pulmonary*[Title/Abstract])) AND (Rehabilitation[Title/Abstract]) AND (VR[Title/Abstract] OR virtual reality[Title/Abstract])

**ScienceDirect**

Results: 19

((covid)[ti,ab,kw ] OR (respiratory)[ti,ab,kw ] OR (pulmonary)[ti,ab,kw ]) AND (rehabilitation)[ti,ab,kw ] AND ((vr)[ti,ab,kw ] OR (virtual reality)[ti,ab,kw ])

**Cochrane**

Results: 0

((Covid):ti,ab,kw OR (COPD):ti,ab,kw OR (respiratory):ti,ab,kw OR (pulmonary):ti,ab,kw) AND (rehabilitation):ti,ab,kw AND (vr OR virtual reality):ti,ab,kw

**CINAHL**

Results: 10

(MH Covid-19 OR pulmonary OR respiratory) AND (TI rehabilitation OR AB rehabilitation) AND (TX vr or virtual reality)

**Web of Science**

Results: 49

(Abstract (Covid) OR (respiratory) OR (pulmonary)) AND Titel Rehabilitation AND (All Fields (vr) OR (virtual reality))

**PEDro**

Results: 3

((Covid):ti,ab OR (COPD):ti,ab OR (respiratory):ti,ab OR (pulmonary):ti,ab,kw) AND (rehabilitation):ti,ab AND (vr OR virtual reality):ti,ab
